# An outbreak of echovirus 18 encephalitis/meningitis in children in Hebei Province, China, 2015

**DOI:** 10.1038/emi.2017.39

**Published:** 2017-06-21

**Authors:** Xiangpeng Chen, Jingjie Li, Jiayun Guo, Wenbo Xu, Suzhen Sun, Zhengde Xie

**Affiliations:** 1 MOE Key Laboratory of Major Diseases in Children, National Key Discipline of Pediatrics, National Clinical Research Center for Respiratory Diseases, Beijing Key Laboratory of Pediatric Respiratory Infectious Diseases, Beijing Pediatric Research Institute, Beijing Children’s Hospital, Capital Medical University, Beijing 100045, China; 2 Department of Neurology, Children’s Hospital of Hebei Province, Shijiazhuang 050031, Hebei Province, China; 3 WHO WPRO Regional Polio Reference Laboratory and Key Laboratory of Medical Virology and Viral Diseases, National Health and Family Planning Commission of China, National Institute for Viral Disease Control and Prevention, Chinese Center for Disease Control and Prevention, Beijing 102206, China

**Dear Editor,**

Viral encephalitis (VE) and viral meningitis (VM) are severe infectious diseases that cause substantial morbidity and mortality in children. Non-polio human enteroviruses (EVs), especially the species Enterovirus B (EV-B), are major causative agents of these diseases. Echovirus 18 (E18), a serotype of EV-B, has mostly been reported as an important pathogen that has caused many outbreaks of aseptic meningitis in the United States,^1, 2^ Japan,^3^ Korea,^4^ Germany,^5^ and other countries or regions.^6^ So far, there has been no report of an E18-associated encephalitis/meningitis outbreak in mainland China, but little E18 epidemiological data is available.^7^ Here we describe an outbreak of E18 encephalitis/meningitis in children that occurred in Hebei Province, China, in 2015.

From April 2015, a marked increase in the number of children with VE/VM was observed in Hebei Province, in the central part of China, whereas only three cases were found from January to March 2015. Therefore, we conducted a study to explore the viral pathogens in these encephalitis/meningitis cases. A total of 268 pediatric VE/VM cases (190 male and 78 female) were enrolled in this study from January to September. The median age of the patients was 5.81 years, with the range being 7 months to 16 years (mean age, 6.11±2.70 years). Most patients (83.6%, 224/268) were 3–10 years old, and no neonates were enrolled in this study. The most predominant clinical manifestations were headache (89.9%, 241/268), vomiting (81.3%, 218/268), fever (62.7%, 168/268), lethargy (26.9%, 72/268) and nausea (22.0%, 59/268), whereas some patients had abdominalgia (9.0%, 24/268), convulsions (3.7%, 10/268) and confusion (2.6%, 7/268). All patients recovered, except for one patient who had secondary epilepsy.

Cerebrospinal fluid (CSF) samples were collected from every case. Viral RNA was extracted from the CSF with a QIAamp MinElute Virus Spin Kit (Qiagen, Hilden, Germany). Viral pathogens, including EVs, Japanese encephalitis virus and mumps virus, were screened using real-time PCR. Herpes viruses, including herpes simplex virus 1 (HSV1), HSV2, varicella zoster virus, Epstein–Barr virus, human herpes virus 6 and cytomegalovirus, were screened by multiple PCR methods with the Seeplex meningitis-V1 ACE Detection kit (Seegene, Seoul, Korea). Partial *VP1* genes from EVs were amplified with degenerate primers.^8, 9^ EV genotyping was done by sequencing the partial *VP1* genes and performing BLAST analysis. The complete *VP1* gene sequences of E18 were obtained by further amplification and sequencing with E18 *VP1*-specific primers. Nucleotide sequence alignments and identities were implemented in MEGA5.03 software (version 5.0; Sudhir Kumar, Arizona State University, Tempe, AZ, USA).^10^ Phylogenetic analysis was conducted on the complete *VP1* gene sequences (this study and GenBank database) by using Bayesian Evolutionary Analysis Sampling Trees (BEAST) version 1.7 software.^11^ Potential recombination of the E18 strain was scanned using a similarity plot and bootscan method with the Simplot program (version 3.5.1; Stuart Ray, Johns Hopkins University, Baltimore, MD, USA).^12^ According to the results of the screening, the EV-positive samples were cultured on rhabdomyosarcoma cell lines for virus isolation. After high titer virus was obtained, a representative strain was selected to sequence the complete genome.

The total positive detection rate of viruses was 64.9% (174/268), EVs accounted for 51.1% (89/174), followed by HSV1 (19.5%, 34/174), varicella zoster virus (10.9%, 19/174), HSV2 (6.3%, 11/174), Epstein–Barr virus (4.6%, 8/174), cytomegalovirus (4.0%, 7/174) and human herpes virus 6 (3.4%, 6/174). No Japanese encephalitis virus and mumps virus-positive samples were detected. The cases peaked in July and August (Supplementary Figure S1). Among the 82 genotyped EVs, E18 was the most predominant, accounting for 74.4% (61/82), followed by E30 (17.1%, 14/82), E6 (3.7%, 3/82), E14 (1.2%, 1/82), E33 (1.2%), coxsackievirus A9 (CV-A9; 1.2%) and CV-B5 (1.2%). The genotypes of seven EVs were not confirmed because of poor sequence data quality. A total of 57 E18 complete *VP1* gene sequences were obtained (accession number KY303773–KY303829). Seventy-one EV-positive CSF samples were cultured with rhabdomyosarcoma cell, and 29 EV strains were obtained, including 18 strains of E18, 8 strains of E30 and 3 strains of E6 (Supplementary Table S1). The remaining 42 CSF samples had negative cultures.

A phylogenetic tree based on the complete *VP1* gene sequences was constructed by the BEAST software (Figure 1). Referring to the genotyping criteria of CV-A16,^13^ more than 15% of the nucleotide difference in the complete *VP1* region of the E18 strain was used to distinguish the genotypes in this study. According to the results, except for the E18 prototype strain (Metcalf, accession number AF317694), all the E18 strains could be divided into three genotypes—A, B and C—with the support of the high confidence values (posterior value >99%). The mean genetic distance between genotypes A and B was 0.208, A and C was 0.245, and B and C was 0.218. The nucleotide identities were 80.1%–83% (A and B), 76.5%–81.4% (A and C) and 79.4%–84.3% (B and C). The prototype Metcalf shared 79.6%–79.7%, 78.9%–80.8% and 77.1%–80.3% nucleotide identity with genotypes A, B and C, respectively. The two strains of genotype A were in China in 2005 and 2011, respectively. Genotype B was collected in India in 2008, and the strains shared 95.8%–98.6% nucleotide identity. Genotype C can be further divided into two subgenotypes (C1 and C2), with the support of high confidence values (posterior value >99%) and a mean genetic distance of 0.138 between the subtypes. All isolates from Hebei Province in 2015 clustered into the C2 subtype. The strains in the C1 subtype shared 90%–100% nucleotide identity and 92.3%–100% amino-acid identity with each other. The strains in the C2 subtype shared 86.7%–100% nucleotide identity and 92.3%–100% amino-acid identity with each other.

The B–C loop, located from amino acid 78–89 in the E18 VP1 region, is important for the reactivity of type-specific neutralizing antibodies, which formed a part of the epitope on the surface of the virion.^14^ There were 18 amino-acid substitutions between the prototype strain and the strains in this study. One amino-acid substitution was located at residue 84 (R→N) in the B-C loop. These amino-acid changes might be a significant cause of the prevalence of E18, but this will require intensive studies.

The complete genome sequence of the E18–314 isolate was available and was analyzed (accession number KX767786). The nucleotide identity of the complete genomes was 80.6% compared with the prototype strain, Metcalf. The nucleotide identities of the *5′UTR, P1, P2, P3, 3′UTR* and *VP1* regions of E18–314 compared with the prototype strain were 81.5%, 80.2%, 79.7%, 81.0%, 87.1% and 80.7%, respectively.^15^ The EV-B prototype strains were used as reference sequences, and similarity plots and bootscanning analyses were performed to depict the possible recombination of the E18 strain. The results showed the highest similarity and nearest phylogenetic relationship with the E18 prototype in the *P1* region. However, in the *5′UTR, P2* and *P3* regions, the E18–314 strain contained some regions that showed higher similarity with the E9, CV-B4, EV-B106, E13 and E16 prototypes, but apparently not the Metcalf strain. Two obvious crossing sites in the *5′UTR* and *2A* regions suggested that a potential recombination occurred (Supplementary Figure S2).

These results indicated that there was an outbreak of E18 encephalitis/meningitis in children in Hebei Province, China, in 2015. The phylogenetic analysis suggests that the outbreak was caused by a new E18 strain. The representative strain, E18–314, was a potential multiple-recombinant virus, which contained other EV-B donor sequences. Genetic recombination is sometimes an important reason for changes in enterovirus virulence and can trigger serious public health problems.

Little data on the molecular epidemiology of E18 in mainland China has been obtained before the current study. Further study is needed to gain a better insight into the prevalence of E18 infection in mainland China.

## Figures and Tables

**Figure 1:**
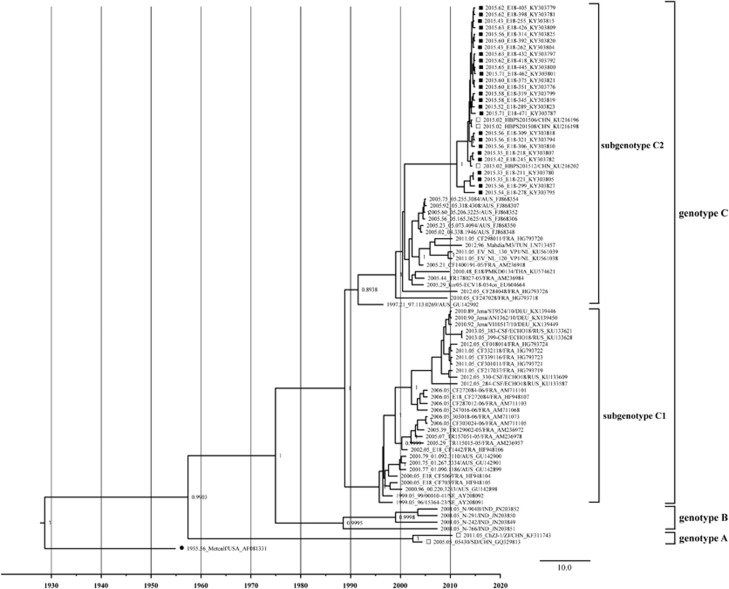
The phylogenetic tree based on the alignments of the complete *VP1* gene sequences of E18 was constructed with BEAST software (100 million generations) using a lognormal relaxed clock, a constant size tree prior and the GTR+G substitution model. Squares indicate China E18, whereas filled squares indicate the sequences from this study. Filled circle indicates the E18 Metcalf prototype strain. The scale bar indicates years. The strain name, year of sampling and GenBank accession numbers are presented.
