# Supplementary material for: An outbreak of echovirus 18 encephalitis/meningitis in children in Hebei Province, China, 2015
Source: Emerg Microbes Infect. 2017 Jun 21;6(6):e54–. doi: 10.1038/emi.2017.39 (PMC5584482; doi:10.1038/emi.2017.39)
Supplement: Supplementary Figure S1 [file emi201739x1.doc]

**Supplementary information**


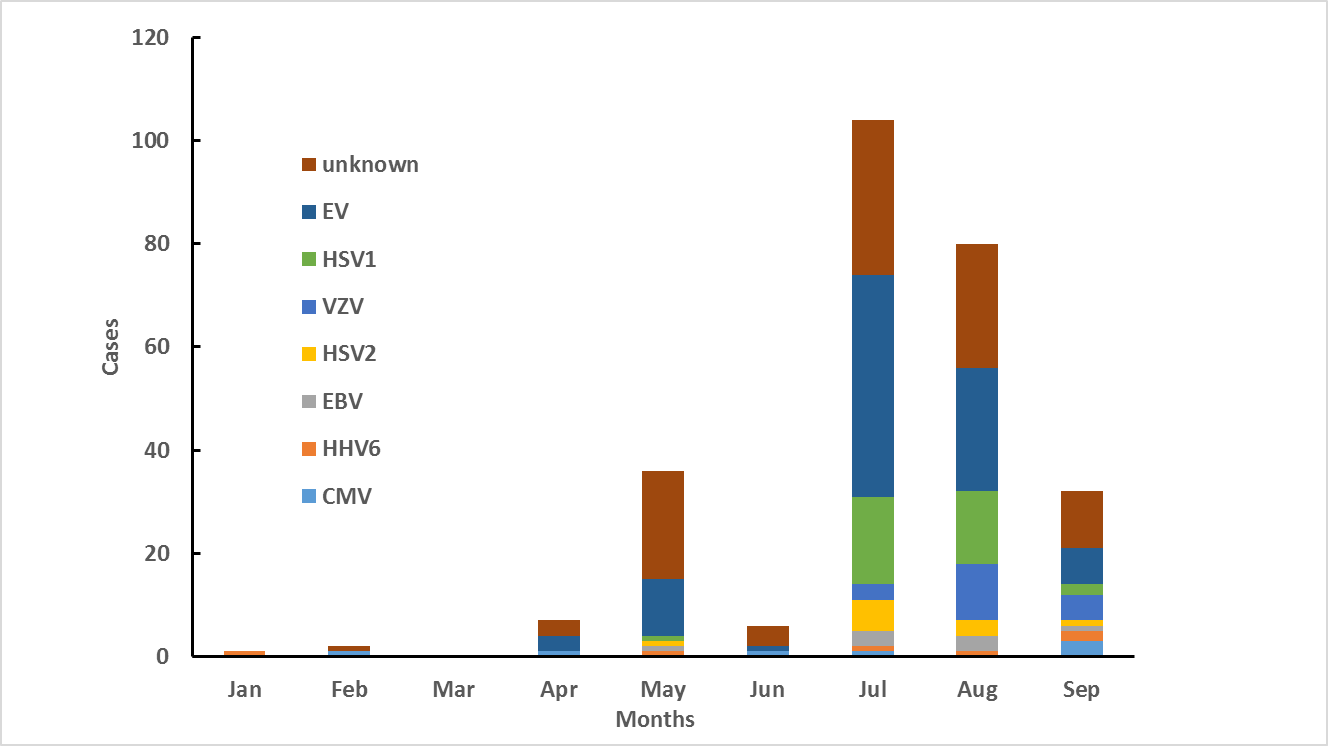


Figure S1. Seasonal distribution of viral encephalitis/meningitis cases and screened viruses in each month.
