# Supplementary material for: An outbreak of echovirus 18 encephalitis/meningitis in children in Hebei Province, China, 2015
Source: Emerg Microbes Infect. 2017 Jun 21;6(6):e54–. doi: 10.1038/emi.2017.39 (PMC5584482; doi:10.1038/emi.2017.39)
Supplement: Supplementary Figure S2 [file emi201739x2.doc]

**Supplementary information**


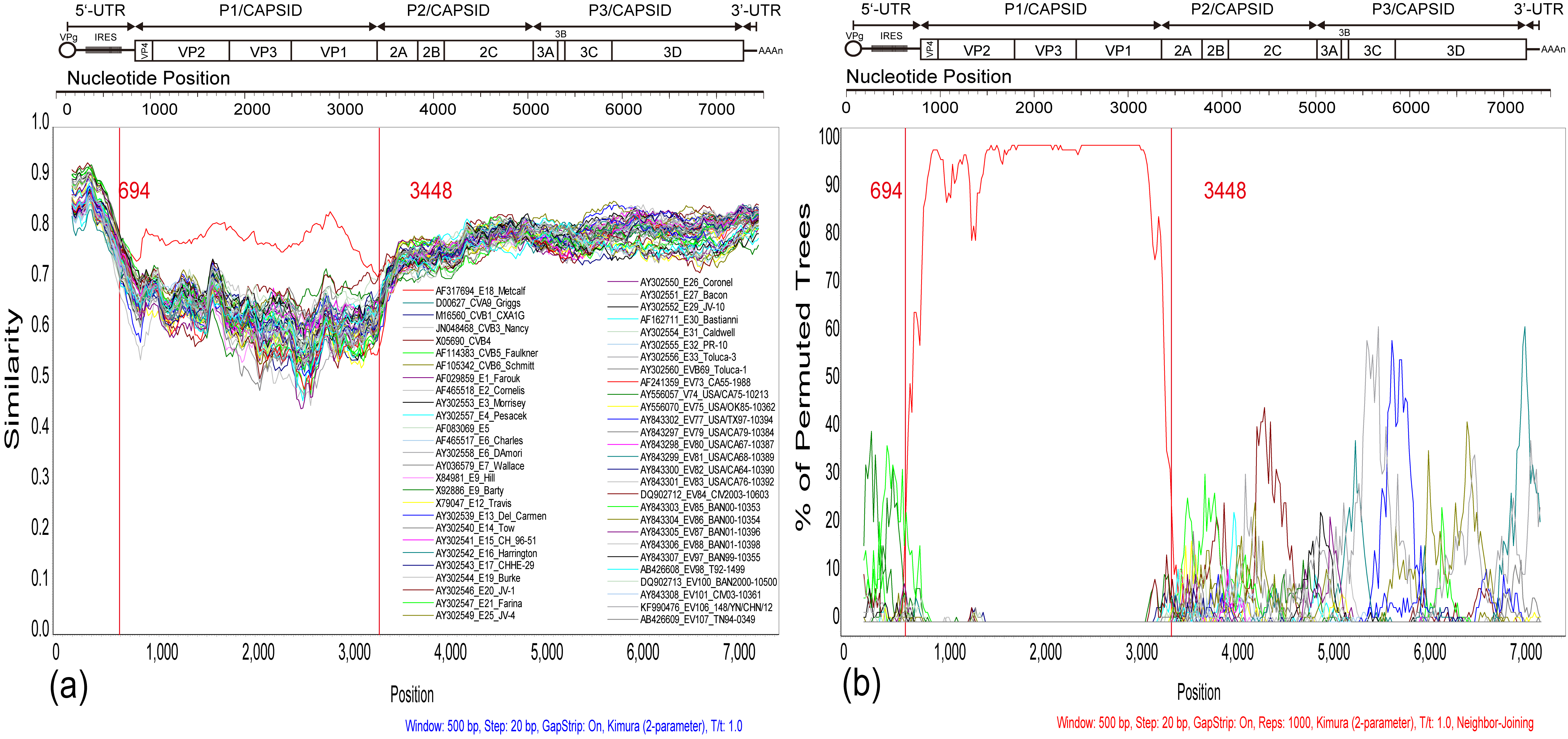


Figure S2. Similarity plot and bootscanning analyses of the E18-314 and other EV-B prototype strains on the basis of full-length genomes. The E18-314 was used as the query sequence. A sliding window of 500 nucleotides moving in 20 nucleotides steps was used in this analysis. The a was the result of similarity plot analyses; the b was the result of bootscanning analyses.
