# Supplementary material for: An outbreak of echovirus 18 encephalitis/meningitis in children in Hebei Province, China, 2015
Source: Emerg Microbes Infect. 2017 Jun 21;6(6):e54–. doi: 10.1038/emi.2017.39 (PMC5584482; doi:10.1038/emi.2017.39)
Supplement: Supplementary Table S1 [file emi201739x3.doc]

Table S1. The clinical and laboratory data of the cases with viral encephalitis/viral meningitis caused by enterovirus

| Case number | Age(ys) | Gender | Diagnosis | EV type found in CSF | Complete VP1 gene (GenBank Acc. No.) | Isolates from CSF samples | |
| --- | --- | --- | --- | --- | --- | --- | --- |
| SJZ-209 | 6.16 | F | VM | E18 |  |  | |
| SJZ-211 | 5.37 | F | VE | E18 | KY303780 |  |  |
| SJZ-215 | 5.55 | M | VE | E18 | KY303775 | E18 | |
| SJZ-218 | 4.47 | M | VE | E18 | KY303807 |  | |
| SJZ-221 | 6.66 | M | VE | E18 | KY303805 | E18 | |
| SJZ-225 | 6.38 | M | VM | Failed |  |  | |
| SJZ-226 | 3.26 | F | VE | Failed |  |  | |
| SJZ-228 | 6.82 | M | VE | Failed |  |  | |
| SJZ-229 | 5.42 | M | VE | E18 | KY303828 |  | |
| SJZ-232 | 3.03 | F | VM | E30 |  | E30 | |
| SJZ-245 | 4.07 | M | VM | E18 | KY303782 | E18 | |
| SJZ-252 | 6.18 | M | VM | E30 |  | E30 | |
| SJZ-255 | 11.81 | F | VM | E18 | KY303815 |  | |
| SJZ-256 | 6.43 | M | VM | E30 |  | E30 | |
| SJZ-262 | 3.38 | F | VE | E18 | KY303804 | E18 | |
| SJZ-265 | 2.64 | F | VE | E18 | KY303773 |  | |
| SJZ-273 | 5.76 | M | VM | E18 | KY303816 |  | |
| SJZ-275 | 7.30 | M | VE | E18 | KY303817 |  | |
| SJZ-278 | 4.39 | M | VM | E18 | KY303795 |  | |
| SJZ-289 | 3.13 | M | VM | E18 | KY303823 | E18 | |
| SJZ-290 | 4.35 | F | VE | Failed |  |  | |
| SJZ-291 | 16.15 | M | VE | E18 | KY303813 | E18 | |
| SJZ-292 | 8.50 | M | VE | E30 |  |  | |
| SJZ-293 | 6.92 | M | VE | E18 | KY303798 |  | |
| SJZ-294 | 8.27 | F | VE | E30 |  | E30 | |
| SJZ-295 | 5.69 | F | VM | E30 |  |  | |
| SJZ-296 | 8.34 | M | VM | E30 |  | E30 | |
| SJZ-297 | 7.01 | F | VE | E30 |  | E30 | |
| SJZ-298 | 3.90 | M | VM | Failed |  |  | |
| SJZ-299 | 6.51 | M | VM | E18 | KY303827 |  | |
| SJZ-301 | 4.49 | F | VE | E18 | KY303788 |  | |
| SJZ-302 | 5.14 | F | VM | E18 | KY303774 | E18 | |
| SJZ-303 | 6.06 | M | VE | E18 | KY303796 |  | |
| SJZ-304 | 9.56 | F | VM | E6 |  | E6 | |
| SJZ-306 | 7.60 | M | VE | E18 | KY303810 | E18 | |
| SJZ-307 | 9.81 | F | VM | E18 | KY303790 |  | |
| SJZ-308 | 8.38 | F | VM | E18 | KY303785 | E18 | |
| SJZ-309 | 9.88 | M | VE | E18 | KY303818 |  | |
| SJZ-311 | 4.25 | M | VE | E18 | KY303803 |  | |
| SJZ-313 | 9.86 | F | VE | E18 | KY303824 |  | |
| SJZ-314 | 4.67 | M | VM | E18 | KY303825 | E18 | |
| SJZ-315 | 5.59 | M | VE | E30 |  |  | |
| SJZ-316 | 8.27 | M | VE | CV-A9 |  |  | |
| SJZ-319 | 5.04 | F | VE | E18 | KY303799 |  | |
| SJZ-321 | 5.11 | M | VE | E18 | KY303794 | E18 | |
| SJZ-324 | 13.16 | M | VM | E30 |  |  | |
| SJZ-327 | 3.01 | M | VE | E30 |  |  | |
| SJZ-328 | 4.04 | M | VM | E33 |  |  | |
| SJZ-344 | 6.08 | M | VE | E18 | KY303793 |  | |
| SJZ-345 | 4.14 | M | VE | E18 | KY303819 | E18 | |
| SJZ-351 | 8.47 | M | VE | E18 | KY303776 |  | |
| SJZ-354 | 3.75 | M | VM | Failed |  |  | |
| SJZ-356 | 2.86 | M | VE | E6 |  | E6 | |
| SJZ-360 | 5.97 | M | VE | E18 | KY303826 |  | |
| SJZ-365 | 8.80 | M | VM | E18 | KY303777 |  | |
| SJZ-366 | 7.06 | F | VE | E6 |  | E6 | |
| SJZ-370 | 6.04 | M | VM | E18 |  |  | |
| SJZ-371 | 3.48 | M | VE | E18 | KY303791 |  | |
| SJZ-372 | 3.75 | M | VE | E18 | KY303783 |  | |
| SJZ-375 | 9.73 | M | VE | E18 | KY303821 |  | |
| SJZ-377 | 5.68 | M | VM | E18 | KY303806 | E18 | |
| SJZ-378 | 2.22 | M | VE | E14 |  |  | |
| SJZ-387 | 8.36 | F | VM | E30 |  | E30 | |
| SJZ-392 | 6.35 | F | VM | E18 | KY303820 |  | |
| SJZ-393 | 9.78 | M | VE | E18 | KY303784 | E18 | |
| SJZ-394 | 3.15 | M | VE | E18 | KY303778 |  | |
| SJZ-398 | 8.53 | F | VE | E18 | KY303781 | E18 | |
| SJZ-405 | 9.40 | M | VE | E18 | KY303779 |  | |
| SJZ-407 | 9.59 | M | VE | E18 | KY303829 |  | |
| SJZ-415 | 9.86 | M | VE | E18 | KY303812 |  | |
| SJZ-417 | 6.21 | M | VM | E18 | KY303802 |  | |
| SJZ-418 | 3.38 | M | VE | E18 | KY303792 |  | |
| SJZ-422 | 5.68 | M | VE | E18 | KY303811 |  | |
| SJZ-423 | 8.91 | M | VM | E18 | KY303814 |  | |
| SJZ-425 | 10.11 | M | VM | E18 | KY303786 |  | |
| SJZ-426 | 9.01 | M | VE | E18 | KY303809 |  | |
| SJZ-432 | 5.54 | F | VE | E18 | KY303797 |  | |
| SJZ-433 | 5.54 | M | VM | E18 | KY303789 |  | |
| SJZ-437 | 2.58 | F | VE | E18 | KY303808 |  | |
| SJZ-438 | 4.89 | F | VM | E18 |  |  | |
| SJZ-443 | 3.00 | M | VE | CV-B5 |  |  | |
| SJZ-445 | 3.67 | M | VE | E18 | KY303800 |  | |
| SJZ-449 | 3.03 | F | VM | Failed |  |  | |
| SJZ-459 | 10.35 | M | VE | E30 |  |  | |
| SJZ-462 | 6.26 | M | VE | E18 | KY303801 | E18 | |
| SJZ-463 | 4.80 | M | VE | E30 |  | E30 | |
| SJZ-464 | 7.13 | M | VE | E18 |  | E18 | |
| SJZ-467 | 6.52 | M | VM | E18 | KY303822 | E18 | |
| SJZ-471 | 13.92 | M | VM | E18 | KY303787 |  | |
